# The effects of ordered carbon vacancies on stability and thermo-mechanical properties of V_8_C_7_ compared with VC

**DOI:** 10.1038/srep34007

**Published:** 2016-09-23

**Authors:** XiaoYu Chong, YeHua Jiang, Rong Zhou, Jing Feng

**Affiliations:** 1 Faculty of Material Science and Engineering, Kunming University of Science and Technology, Kunming 650093, People’s Republic of China

## Abstract

The ordered non-stoichiometric V_8_C_7_ can form in the VC_y_ carbides by the disorder–order phase transformation. The intrusion of ordered carbon vacancies can affect their stability, mechanical, thermal and electronic properties. The relatively thermodynamic stability and mechanical properties at high temperature for the ordered stoichiometric VC and non-stoichiometric V_8_C_7_ are investigated in this paper by first-principle calculations combined with the quasi-harmonic approximation. The difference between the properties of VC and V_8_C_7_ can be obtained. We find that the V_8_C_7_ is thermodynamic more stable than VC, but has weaker elastic heat resistance than VC. Moreover, the minimum thermal conductivity of VC is a little larger than V_8_C_7_ and a simple way is proposed to characterize the anisotropy of lattice thermal conductivity based on the Cahill’s model.

Vanadium carbides display a unique combination of thermal and mechanical properties such as high hardness, high melting point, high-temperature strength, corrosion and wear resistance and very efficient electrical and thermal conductivities. So they are widely used in functional coatings, corrosion protection, high-temperature structural materials, microelectronics and catalysts. Vanadium form the stoichiometric carbides such as VC and the non-stoichiometric cubic monocarbide VC_y_ which are all with B1 (NaCl) structure. A feature peculiar to the structure of non-stoichiometric VC_y_ is the intrusion of carbon atoms in the octahedral interstices of the metallic sites and the carbon atoms may occupy only a fraction of the interstitial sites, and the rests are filled with structural vacancies. The carbon-atom vacancies arranging randomly on the fcc carbon sublattice is called disordered phases, while the ordered carbon vacancies take on a periodic arrangement. The disordered state of non-stoichiometric carbides is in thermodynamic equilibrium only at a high temperature, whereas the ordered state is in thermodynamic equilibrium at a temperature below 1300 K^1^. The disorder–order phase transition in the vanadium carbides and a cubic ordered V_8_C_7_ phase can arise in the VC_y_ carbide over the range VC_0.86_–VC_0.88_^2^,^3^. The low-temperature phase V_8_C_7_ does not have an fcc structure, it crystallizes in the space group P4_3_32 with the vanadium atoms occupying positions slightly off the ideal fcc positions and the carbon atoms having an ordered arrangement on the interstitial sites^4^. The crystal structures of VC and V_8_C_7_ are presented in Fig. 1. The existence of ordered carbon vacancies can affect the stability, mechanical, thermal and electronic properties of vanadium carbides. The influence of the order-disorder transformation on the electrical resistivity of single crystals of vanadium carbide was studied by Shacklette and Williams^4^. They found that the electrical resistivity of the disordered vanadium carbide is significantly higher than that of ordered phase and the temperature of order–disorder transformation is 1124 ± 15 °C for V_8_C_7_. Emmons and Williams studied the thermodynamics of δ-VC_1−x_ order-disorder transformation using powder samples^5^. Lipatnikov’s research results show that ordered structure produces much higher hardness and electrical conductivity^6^. They also investigated the disorder–order phase transformations in the region of homogeneity of a non-stoichiometric cubic vanadium carbide, VC_y_ (0.66 < y < 0.88) and constructed the equilibrium phase diagram of the V–C system which allows for the formation of ordered phases in a non-stoichiometric vanadium carbide^1^. Xing *et al*. reported a new type vanadium carbide V_5_C_3_ and predicted its mechanical properties^7^. In our previous work, we calculated the mechanical and thermal properties of the ordered vanadium carbides system including VC, V_2_C, V_4_C_3_, V_6_C_5_ and V_8_C_7_^8^. But the difference for the thermodynamic and mechanical properties of ordered vanadium carbides have not been reported so for. This work contains two aspects. One is the difference for relatively thermodynamic stability and mechanical properties for the ordered stoichiometric VC and non-stoichiometric V_8_C_7_ at high temperature are obtained and compared with the theoretical and experimental results. The other one is a simple way is proposed to characterize the anisotropy of lattice thermal conductivity based on the Cahill’s model.

## Results and Discussion

### Crystal structure

**Figure 2:**
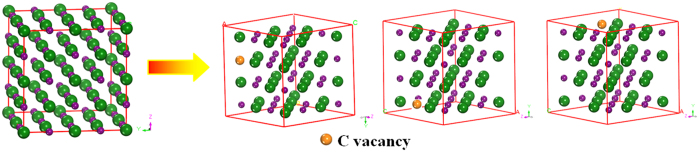
The crystal structure of VC and the ordered carbon vacancy site in V_8_C_7_.

**Figure 3:**
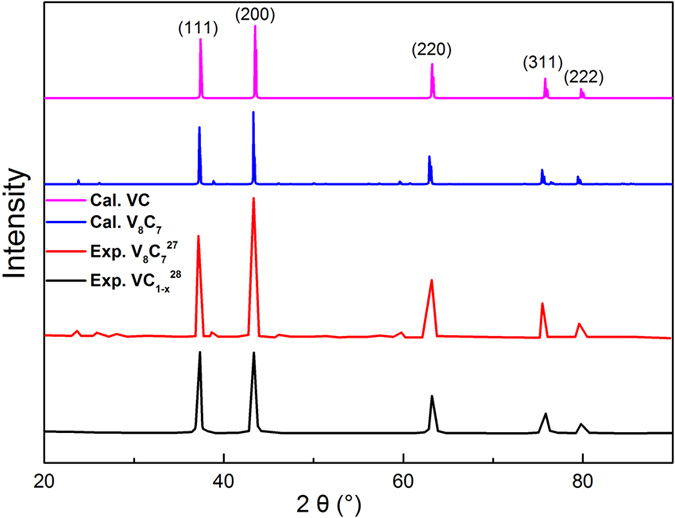
The simulated XRD of VC and V_8_C_7_. The results are compared with the experimental results of V_8_C_7_ and VC_1−x_^9^,^10^.

Figure 2 exhibits the carbon vacancy site in V_8_C_7_ which is not an fcc structure. Details for the structure of VC and V_8_C_7_ can be seen in Table S1. By comparing the simulated XRD of VC and V_8_C_7_ with the experimental results of V_8_C_7_ and VC_1−x_, we find our simulated results are in agreement with the experimental results^9^,^10^. And from the XRD pattern shown in Fig. 3, it is difficult to distinguish the ordered VC, V_8_C_7_ and disordered VC_1−x_. So we investigate the difference of the mechanical and thermal properties of them under different temperature.

### Thermodynamic properties

**Figure 4:**
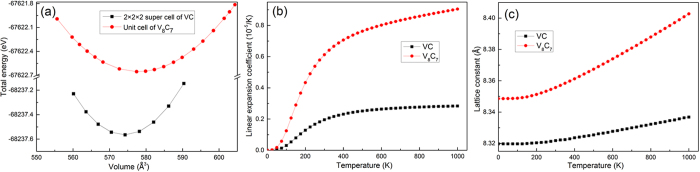
(**a**) The curve of the volume dependence of total energy; (**b**) The linear expansion coefficients of VC and V_8_C_7_ at different temperature; (**c**) Lattice constant of V_8_C_7_ and 2 × 2 × 2 supercell of VC as a function of temperature.

The calculated results of thermal properties from Helmholtz free energy at elevated temperatures are showing in Fig. 4. From Fig. 4(b), we can see that the linear expansion coefficient of V_8_C_7_ is larger than VC during the temperature from 0 K to 1000 K, which leads to the Lattice constant of V_8_C_7_ increases more rapidly than the 2 × 2 × 2 supercell of VC with the temperature increasing (Fig. 4(c)).

**Figure 5:**
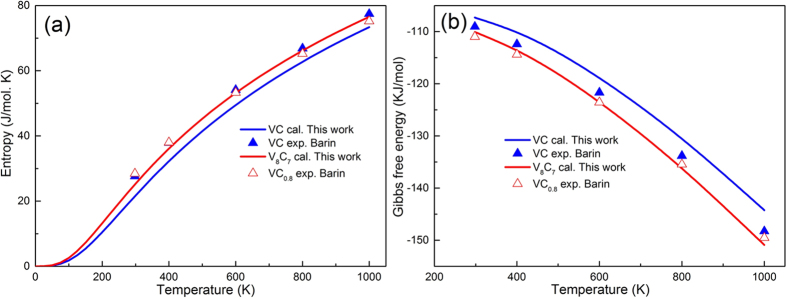
(**a**) The entropy (*S*) of VC and V_8_C_7_ as a function of temperature; (**b**) The Gibbs free energy (*G*) of VC and V_8_C_7_ as a function of temperature companied with the experimental data^11^.

Moreover, the obtained thermodynamic parameters, such as entropy (*S*) and Gibbs free energy (*G*), of VC and ordered V_8_C_7_ as a function of temperature exhibit in Fig. 5, which are in good agreement with the experimental results^11^. From Fig. 5(a), the entropy of V_8_C_7_ is larger than the entropy of VC, indicating that the V_8_C_7_ is thermodynamic more stable than VC at different temperature. The result can be confirmed by the Gibbs free energy of VC and V_8_C_7_ as shown in Fig. 5(b). The calculated Gibbs free energy of V_8_C_7_ is smaller than that of VC, which also suggests that the V_8_C_7_ is more stable at high temperature and easier to form than VC. What’s more, there is a little error between the experimental and theoretical data of entropy and Gibbs energy of VC, which can attribute to the experimental conditions and magnetic contribution to the entropy and Gibbs energy.

**Figure 6:**
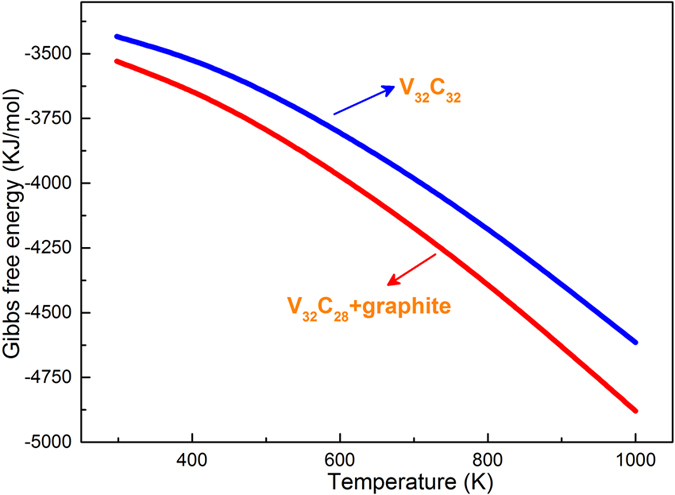
The Gibbs free energy of V_32_C_32_ and the V_32_C_28_ + graphite system as a function of temperature.

The further analysis for the relatively thermodynamic stability of VC and V_8_C_7_ can be seen in Fig. 6. The Gibbs free energy of V_32_C_32_ is larger than the system of V_32_C_28_ and graphite, which shows that the V_32_C_32_ tends to decompose into V_32_C_28_ and graphite from the room temperature to 1000 K. And the result supports that the introduction of carbon vacancies in the B1 crystal structure and different symmetry increase the stability of carbides^12^. This founding explains the phenomenon that vanadium carbides phases in the high-vanadium iron and steel are not the stoichiometric compounds VC, but always the non-stoichiometric VC_1−x_ such as V_8_C_7_ and V_4_C_3_^13^.

### Elastic properties at finite temperature

**Figure 7:**
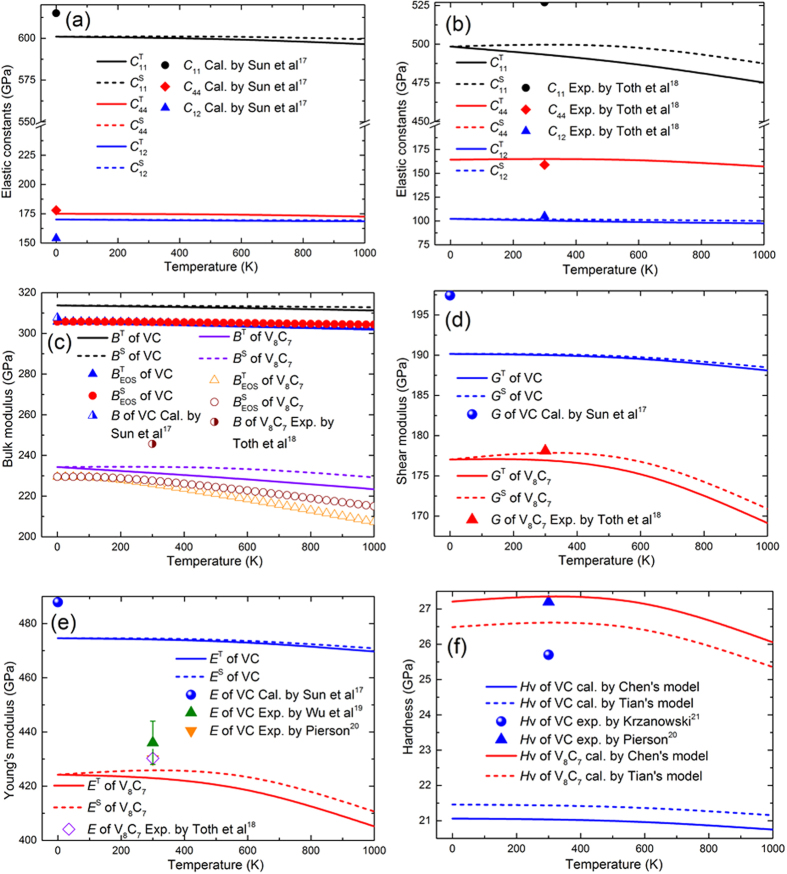
Temperature dependent elastic constants, mechanical modulus and intrinsic hardness of VC and V_8_C_7_. (**a**) Elastic constants of VC; (**b**) Elastic constants of V_8_C_7_; (**c**) Bulk modulus of VC and V_8_C_7_; (**d**) Shear modulus of VC and V_8_C_7_; (**e**) Young’s modulus of VC and V_8_C_7_; (**f**) Intrinsic hardness of VC and V_8_C_7_.

The obtained isothermal and isentropic elastic constants of VC and ordered V_8_C_7_ as a function of temperature from 0 K to 1000 K are displayed in Fig. 7(a,b). Different decreased amplitudes of 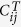 and 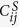 values with the increase of temperature could be found for VC and V_8_C_7_. It can be seen clearly that the elastic constants of VC decrease slightly and approach zero slope with the increase of temperature. The reduction in elastic constants values is more pronounced for V_8_C_7_ than VC, and that such a decrease of *C*_11_ with temperature is more apparent. The value of 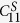 is larger than 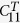 for V_8_C_7_ and the deviation of our calculated results to experimental data for 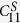 of V_8_C_7_ is about 5%.

**Figure 8:**
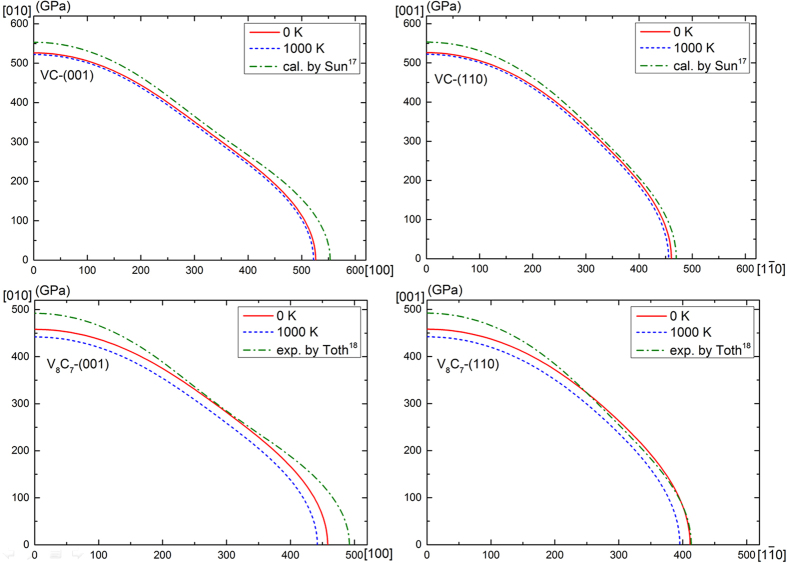
The planner projections of the anisotropy of Young’s modulus of VC and V_8_C_7_ on (001) and (110) planes at different temperatures.

Using the elastic constants, the aggregate properties of polycrystalline such as the isothermal and isentropic bulk modulus (*B*^T^ and *B*^S^), shear modulus (*G*^T^ and *G*^S^) and Young’s modulus (*E*^T^ and *E*^S^) of VC and V_8_C_7_ are calculated by VRH approach at different temperature. Moreover, the bulk modulus can also be obtained by the thermal equation of states (*EOS*)^14^:


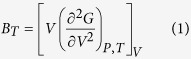



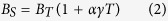


where *γ* is the Grüneisen parameter, *α* is the thermal expansion coefficient and *V* is the equilibrium volume. The results are shown in Fig. 7(c–e). It can be found that the modulus values are larger for VC than those for V_8_C_7_, which is in according with the previous results. Apparently, the mechanical modulus all decease when temperature increases, and an almost linear decrease could be discerned above 400 K. The response of mechanical properties of polycrystalline to the temperature is quite similar to that of elastic constants of single crystal. The isentropic modulus is larger than the isothermal one for VC and V_8_C_7_. And the decrease of mechanical modulus of V_8_C_7_ is more obvious than VC with the temperature increasing. In addition, the intrinsic hardness (*H*_V_) at different temperature is calculated by Chen’s and Tian’s models^15^,^16^,^17^,^18^ and the results are shown in Fig. 8(f). The intrinsic hardness obtained by Tian’s model is higher than that by Chen’s model for VC, but this is opposite for V_8_C_7_. All the two models describe the hardness with the elastic parameters of materials. But there is an extra intercept term (−3) in Chen’s model compared with Tian’s model. Indeed, proper hardness model should be selected according to the chemical bonding characteristics of the crystal. The predicted hardness of V_8_C_7_ is higher than VC but reduce pronouncedly at high temperature. It is known that a material would be less elastic heat resistive if its elastic properties are more sensitive to temperature. So these results indicate that the elastic heat resistant property of VC is superior to those of V_8_C_7_. Our theoretical results deviate from other calculated and experimental values of VC and V_8_C_7_ by less than 3% for *B*^S^ and *G*^S^. The slight discrepancy may be attributed to the ignorance of the anharmonic effect and electron and maganetic contributionon on the kinetic energy. However, the deviation of our calculated results to experimental data for *E*^S^ and *H*_V_ of VC is larger than 10%, which may be caused by the relative strong anisotropy of Young’s modulus. The anisotropy of Young’s modulus for VC and V_8_C_7_ has been demonstrated and investigated in our previous work^8^. Another important reason is that the single crystal of vanadium carbide in the high vanadium iron and steel grows dependent on the orientations and the morphology of them is always dendritic as reported in ref. 19. So the characterized results of Young’s modulus and hardness are related to the location of the vanadium carbide^20^,^21^.

The anisotropy of the Young’s modulus for VC and V_8_C_7_ can be evaluated by calculating the Young’s modulus as a function of crystallographic orientation. The expressions for Young’s modulus in the three principal directions [100], [010] and [001] for cubic crystal is given as^22^:


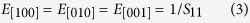


Moreover, in [1 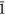 0] and [1 1 1] directions, they are calculated by:


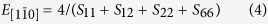



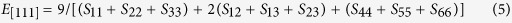


Where *S*_*ij*_ represents the elastic compliances matrices, which can be calculated directly from 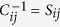. The temperature-dependent *E*^*S*^ is plotted in polar coordinates and projected onto the plane as shown in Fig. 8. For VC and V_8_C_7_, the curve on (001) plane with maximum values along [100] direction and minimum values along the [110] direction could be observed, while on (110) plane, the maximum values and minimum values could be found along [001] and [1
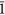
0] directions, respectively. Furthermore, our calculated anisotropy of Young’s modulus at 0 K and 1000 K are all a little weaker than other theoretical and experimental results.

### Minimum lattice thermal conductivity

**Figure 9:**
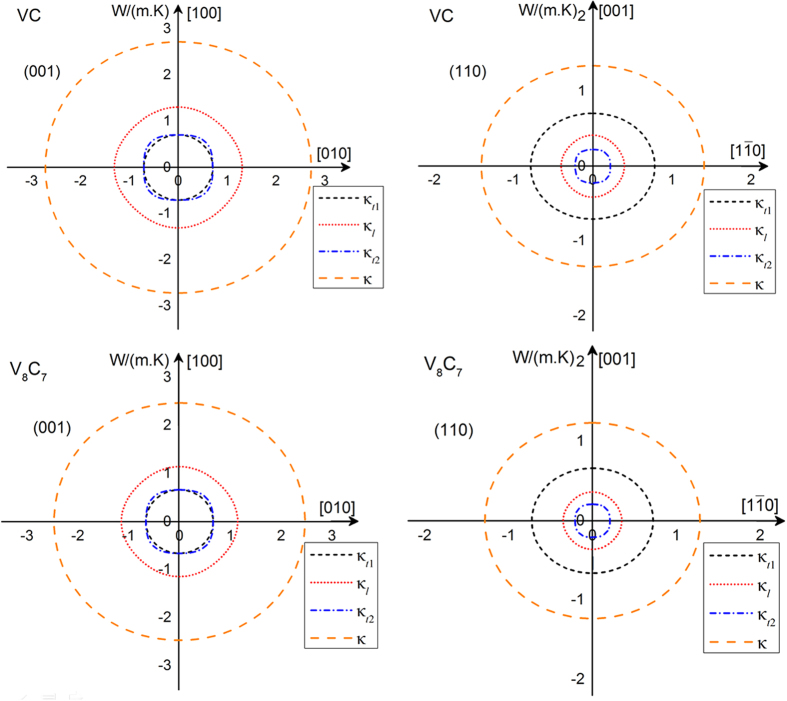
Anisotropy in thermal conductivity of VC and V_8_C_7_ at (001) and (110) crystallographic planes.

This section we will discuss the anisotropy of lattice thermal conductivity for VC and V_8_C_7_ based on Cahill’s model^23^:


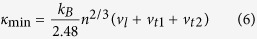


where *n* is the density of number of atoms per volume, and *v*_*l*_, *v*_*t*1_ and *v*_*t*2_ are the longitudinal and transverse modes, respectively. Cahill’s model is suitable for studying the anisotropy of thermal conductivity, because the model already treats the total thermal conductivity as the summation of three acoustic branches. Detailed method for calculating the longitudinal and transverse modes can be found in ref. 24. The results are plotted for VC and V_8_C_7_ on the (001) and (110) crystallographic planes in Fig. 9. One finds that the anisotropy in the calculated thermal conductivities *k*_*l*_ due to *v*_*l*_ is stronger than that of *v*_*t*1_ and *v*_*t*2_. The shapes of these planar contours are related to the anisotropy in elasticity. At (001) crystallographic plane the most contribution to total thermal conductivities is from *k*_*l*_, while at (110) crystallographic plane it is *k*_*t1*_. The most intriguing conclusion obtained from these plots is that the total lattice thermal conductivity (*k*) of VC is almost isotropic. And the thermal conductivity of VC is a little larger than V_8_C_7_, which means that the carbon vacancy or defect can decrease the intrinsic lattice thermal conductivity.

It is known that the more strict treatment of the anisotropy in lattice thermal conductivity of single-crystalline involves the computation of phonon dispersions and applying the relaxation time approximation for phonon scattering. And another way to reveal the anisotropy in thermal conductivity is to evaluate the anisotropy in the Grüneisen constant. In this paper, we provide a simpler way to characterize the anisotropy of lattice thermal conductivity.

## Conclusions

The intrusion of ordered carbon vacancies in non-stoichiometric vanadium carbides can significantly affect their stability, mechanical, thermal and electronic properties. The relatively thermodynamic stability, mechanical properties at high temperature and minimum lattice thermal conductivity of VC and ordered V_8_C_7_ are investigated by first-principle calculations combined with the quasi-harmonic approximation. V_8_C_7_ is more thermodynamically stable at high temperature and easier to form than VC according to the entropy and Gibbs free energy. The isothermal and isentropic elastic constants, mechanical modulus and intrinsic hardness are calculated and we found that the elastic properties of V_8_C_7_ are more sensitive to temperature than VC, which meaning that the elastic heat resistant properties of VC is superior to those of V_8_C_7_. The anisotropic Young’s modulus of VC and V_8_C_7_ are evaluated by calculating the Young’s modulus as a function of crystallographic orientation. Finally, the minimum lattice thermal conductivity of VC and V_8_C_7_ is predicted and a simple way to characterize the anisotropy of lattice thermal conductivity is proposed based on the Cahill’s model.

## Methods

### First-principle calculations details

In this work, all the calculations are performed based on the density functional perturbation theory (DFPT) as implemented in the CASTEP code^25^,^26^. A generalized gradient approximation (GGA) approach in the form of Perdew–Burke–Ernzerhof (PBE) was used to calculate the exchange and correlation functional^27^. The interactions between the ionic cores and the valence electrons were indicated by Ultrasoft pseudo-potentials (UPPs). A plane wave expansion method was applied for the optimization of the crystal structure. A special k-point sampling in the first irreducible Brillouin zone was confirmed by the Monkhorst–Pack scheme, and the k point mesh was selected as 10 × 10 × 10. A kinetic energy cut-off value of 400.0 eV was used for the plane wave expansion in reciprocal space. So the selected values are suitable for the chosen system. The Broyden–Fletcher–Goldfarb–Shannon (BFGS) method was applied to relax the whole structure based on total energy minimization. The total energy changes during the optimization processes were finally converged to 1 × 10^−6^ eV and the forces per atom were reduced to 0.05 eV·Å^−1^. The second-order elastic constants (*C*_11_, *C*_22_ and *C*_44_) at the equilibrium volumes are calculated using an efficient stress–strain method, which was consisted of applying several different types of Lagrangian strains on crystals and calculated Cauchy stress tensor for each strain after optimizing the internal degrees of freedom^28^. Then the Voigt–Reuss–Hill approach was used to calculate the bulk (*B*), shear (*G*) and Young’s modulus (*E*) for polycrystalline crystals^29^.

### Calculations of the thermodynamic properties

The thermodynamic properties of VC and V_8_C_7_ can be calculated by means of the quasi-harmonic approximation and thermal electronic excitation. For a system with the given volume (*V*) and temperature (*T*), the Helmholtz free energy *F* (*V*, *T*) is obtained through combining vibrational *F*_vib_ (*V*, *T*) and electronic *F*_el_ (*V*, *T*) free energy as follows^30^,^31^,^32^.


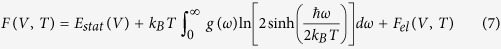


where *E*_stat_(*V*) is the static energy at 0 K and volume *V*. *g* (*ω*, *V*) is the phonon density of states at phonon frequency *ω* and volume *V*. Moreover, the entropy (*S* (*V*, *T*)) is calculated by the vibrational and thermal electronic contribution (*S*_el_ (*V*, *T*))^33^,^34^.


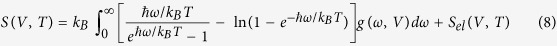


Finally, the temperature dependent equilibrium volume *V* (*T*) and Gibbs free energy *G* (*T*) are obtained by minimization of the following expression^35^ using the thermal equation of states (EOS)^14^:


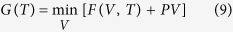


From the results of equilibrium volume *V* (*T*), the volume coefficient of thermal expansion (CTE) *α*_V_ can be calculated by the following equation^36^,^37^:


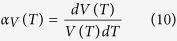


For the cubic crystal, the linear expansion coefficient (*α*_*l*_) and volumetric coefficient (*α*_*V*_) are related by *α*_*V*_ = 3*α*_*l*_.

### Calculation of the temperature-dependent mechanical properties

The temperature-dependent isothermal elastic properties can be estimated by a quasistatic approach, which is assumed that the change of elastic properties is mainly caused by volume change and the anharmonic effect as well as the contributions due to the kinetic energy and the fluctuation of microscopic stress tensors are ignored^38^,^39^. The procedure for calculating the temperature-dependent isothermal elastic constants (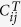) can be summarized in three steps as follows: (i) calculating the static elastic constants at 0 K as a function of volume *C*_*ij*_ (*V*) using the stress-strain energy method; (ii) predicting the volume change as a function of temperature at ambient pressure *V* (*T*, 0) using the quasiharmonic approximation^40^; (iii) then the temperature dependence of isothermal elastic constants can be derived as *C*_*ij*_ (*T*) = *C*_*ij*_ (*T*(*V*)).

Most of the experimental data of elastic stiffness constants are usually reported as isentropic elastic stiffness constants, therefore the calculated isothermal elastic stiffness constants (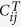) must be converted to the isentropic ones (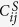) with their thermodynamic relationship given by Davies^41^. For cubic crystals, the thermodynamic relations can be simplified as^42^:


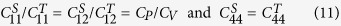


where *C*_V_ and *C*_P_ are heat capacities at constant volume and pressure, respectively. Details about the estimations of *C*_V_ and *C*_P_ from Helmholtz energy and the results can be found in our previous work^8^.

## Additional Information

**How to cite this article**: Chong, X. Y. *et al*. The effects of ordered carbon vacancies on stability and thermo-mechanical properties of V_8_C_7_ compared with VC. *Sci. Rep*. **6**, 34007; doi: 10.1038/srep34007 (2016).

## Supplementary Material

Supplementary Information

## Figures and Tables

**Figure 1:**
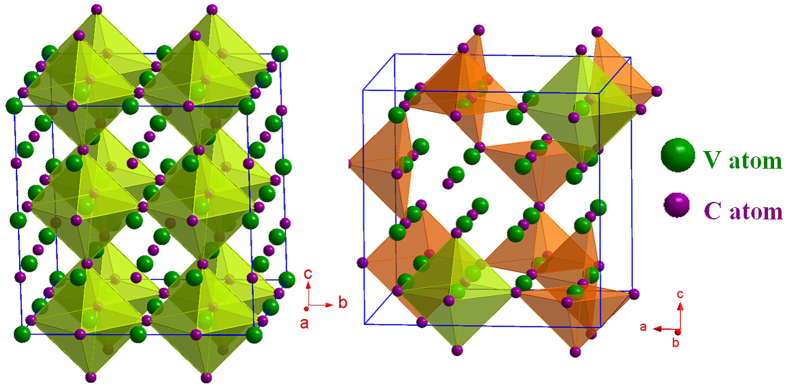
Crystal structure of VC and V_8_C_7_. (**a**) 2 × 2 × 2 supercell of VC with 32 vanadium atoms and 32 carbon atoms; (**b**) Unit cell of V_8_C_7_ with 32 vanadium atoms and 28 carbon atoms.
