# Supplementary material for: The effects of ordered carbon vacancies on stability and thermo-mechanical properties of V8C7 compared with VC
Source: Sci Rep. 2016 Sep 23;6:34007. doi: 10.1038/srep34007 (PMC5034276; doi:10.1038/srep34007)
Supplement: Supplementary Information [file srep34007-s1.pdf]

Supporting Information

The effects of ordered carbon vacancies on stability and thermo-mechanical properties of V_8C_7 compared with VC

XiaoYu Chong, YeHua Jiang, Rong Zhou and Jing Feng*

Faculty of Material Science and Engineering, Kunming University of Science and Technology, Kunming 650093, People's Republic of China

Table S1 Structural information for ordered VC and V_8C_7

Phase	VC	V_8C_7
Space group	Fm-3m	$P4_332$
Z	4	4
Volume (Å^3)	72.25	582.18
Density (g/cm^3)	5.79	5.61
a (Å)	4.165	8.350
V Wyckoff site	4a (0, 0, 0)	8c (0.375, 0.375, 0.375)
		24e (0.125, 0.375, 0.125)
C Wyckoff site	4b (0.5, 0.5, 0.5)	4a (0.125, 0.125, 0.125)
		12d (0.125, 0.625, 0.625)
		12d (0.125, 0.375, 0.875)

Corresponding author: Tel:+1 6174964295, Fax:+1 857-259-2445
E-mails address: jingfeng@kmust.edu.cn
